# Identification of a Tsetse Fly Salivary Protein with Dual Inhibitory Action on Human Platelet Aggregation

**DOI:** 10.1371/journal.pone.0009671

**Published:** 2010-03-23

**Authors:** Guy Caljon, Karin De Ridder, Patrick De Baetselier, Marc Coosemans, Jan Van Den Abbeele

**Affiliations:** 1 Unit of Entomology, Institute of Tropical Medicine Antwerp (ITM), Antwerp, Belgium; 2 Unit of Cellular and Molecular Immunology, Vrije Universiteit Brussel (VUB), Brussels, Belgium; 3 Department of Molecular and Cellular Interactions, Vlaams Instituut voor Biotechnologie (VIB), Ghent, Belgium; Agency for Science, Technology and Research (A*STAR), Singapore

## Abstract

**Background:**

Tsetse flies (*Glossina* sp.), the African trypanosome vectors, rely on anti-hemostatic compounds for efficient blood feeding. Despite their medical importance, very few salivary proteins have been characterized and functionally annotated.

**Methodology/Principal Findings:**

Here we report on the functional characterisation of a 5′nucleotidase-related (5′Nuc) saliva protein of the tsetse fly *Glossina morsitans morsitans*. This protein is encoded by a 1668 bp cDNA corresponding at the genomic level with a single-copy 4 kb gene that is exclusively transcribed in the tsetse salivary gland tissue. The encoded 5′Nuc protein is a soluble 65 kDa glycosylated compound of tsetse saliva with a dual anti-hemostatic action that relies on its combined apyrase activity and fibrinogen receptor (GPIIb/IIIa) antagonistic properties. Experimental evidence is based on the biochemical and functional characterization of recombinant protein and on the successful silencing of the *5′nuc* translation in the salivary gland by RNA interference (RNAi). Refolding of a 5′Nuc/SUMO-fusion protein yielded an active apyrase enzyme with K_m_ and V_max_ values of 43±4 µM and 684±49 nmol P*i*/min×mg for ATPase and 49±11 µM and 177±37 nmol P*i*/min×mg for the ADPase activity. In addition, recombinant 5′Nuc was found to bind to GPIIb/IIIa with an apparent K_D_ of 92±25 nM. Consistent with these features, 5′Nuc potently inhibited ADP-induced thrombocyte aggregation and even caused disaggregation of ADP-triggered human platelets. The importance of 5′Nuc for the tsetse fly hematophagy was further illustrated by specific RNAi that reduced the anti-thrombotic activities in saliva by approximately 50% resulting in a disturbed blood feeding process.

**Conclusions/Significance:**

These data show that this 5′nucleotidase-related apyrase exhibits GPIIb/IIIa antagonistic properties and represents a key thromboregulatory compound of tsetse fly saliva.

## Introduction

Efficient acquisition of a blood meal by hematophagous arthropods relies on a broad repertoire of physiologically active saliva components inoculated at the blood feeding site. These are primarily anti-hemostatic components that interfere with host responses such as vasoconstriction [1], [2], primary hemostasis through the adherence and aggregation of thrombocytes [3], [4] and a secondary hemostatic cascade mainly relying on serine proteases such as thrombin [5], [6]. In the formation of the primary hemostatic plug, adenosine-5′-triphosphate (ATP) and adenosine-5′-diphosphate (ADP) released from injured cells and activated platelets, interact with purinergic P2 receptors (P2X_1_ for ATP and P2Y_1_ and P2Y_12_ for ADP) and fulfil key roles in the activation and aggregation of platelets and strongly contribute to the amplification of the initial hemostatic response [reviewed in [7], [8]]. ADP acts on the G protein-coupled P2Y_1_ and P2Y_12_ receptors resulting in thrombocyte shape change, aggregation (through activation of the fibrinogen receptor) and the production of thromboxane A_2_ that has prothrombotic properties. ATP binds onto the cation-gated P2X_1_ receptor that is present on thrombocytes resulting in cytoskeletal reorganisation and higher responsiveness in terms of aggregation and degranulation towards other platelet activating triggers such as collagen [reviewed in [7], [8]]. To overcome these ATP- and ADP-related host responses, blood sucking insects have ATP/ADP-hydrolysing [ATP(D)ase] enzymes present in their salivary secretions [9]. These enzymes are often called apyrases (nucleosidetriphosphate diphosphohydrolases) and have been described in the saliva of a variety of blood feeding arthropods such as *Cimex lectularius* bed bugs [10], *Ixodes dammini* ticks [11], *Aedes aegypti* and *Anopheles gambiae* mosquitoes [12], [13], *Phlebotomus papatasi* sand flies [14] and *Triatoma infestans* reduviids [15]. These apyrases were shown to mainly belong to two different genetic families: one group of apyrases belongs to the 5′-nucleotidase gene family that has been described in several hematopaghous arthropod species [13], [15], while a completely different type of apyrases has been found in bed bugs (*Cimex* sp.) and sand flies [10], [14], [16]. However, all these apyrases are cation-dependent enzymes that release inorganic phosphate (P*_i_*) from ATP and ADP but not from AMP, thereby inhibiting the purinergic activation and subsequent aggregation of thrombocytes [9], [11]. Beside their thromboregulatory role, apyrases present in the saliva of blood feeding arthropods have also been implicated in anti-inflammation by the degradation of ATP as agonist of the inflammatory purinergic receptors [17]. As such, salivary apyrases of hematophagous arthropods could inhibit several aspects of hemostasis and inflammation thereby promoting the blood feeding process and possibly facilitating pathogen transmission, a feature that we have described for the saliva of tsetse flies, blood feeding insects that transmits the protozoan agents of African trypanosomiasis [18].

In the tsetse fly, the presence of salivary ATP(D)ases has been suggested by the observation that ADP-induced thrombocyte aggregation is inhibited by an unidentified apyrase activity of >30kDa [4]. Strikingly, tsetse saliva could also disaggregate ADP-triggered platelets which was attributed by the authors to the apyrase enzymatic activity. In this study, we report on the identification of a 5′nucleotidase-related protein in *Glossina morsitans morsitans* and provide molecular, biochemical and functional evidence that this is the major apyrase in tsetse saliva that is also able to bind to the fibrinogen receptor and inhibit and reverse ADP-induced platelet responses.

## Results

### *In silico* analysis of the 5′Nuc cDNA

**Figure 1:**
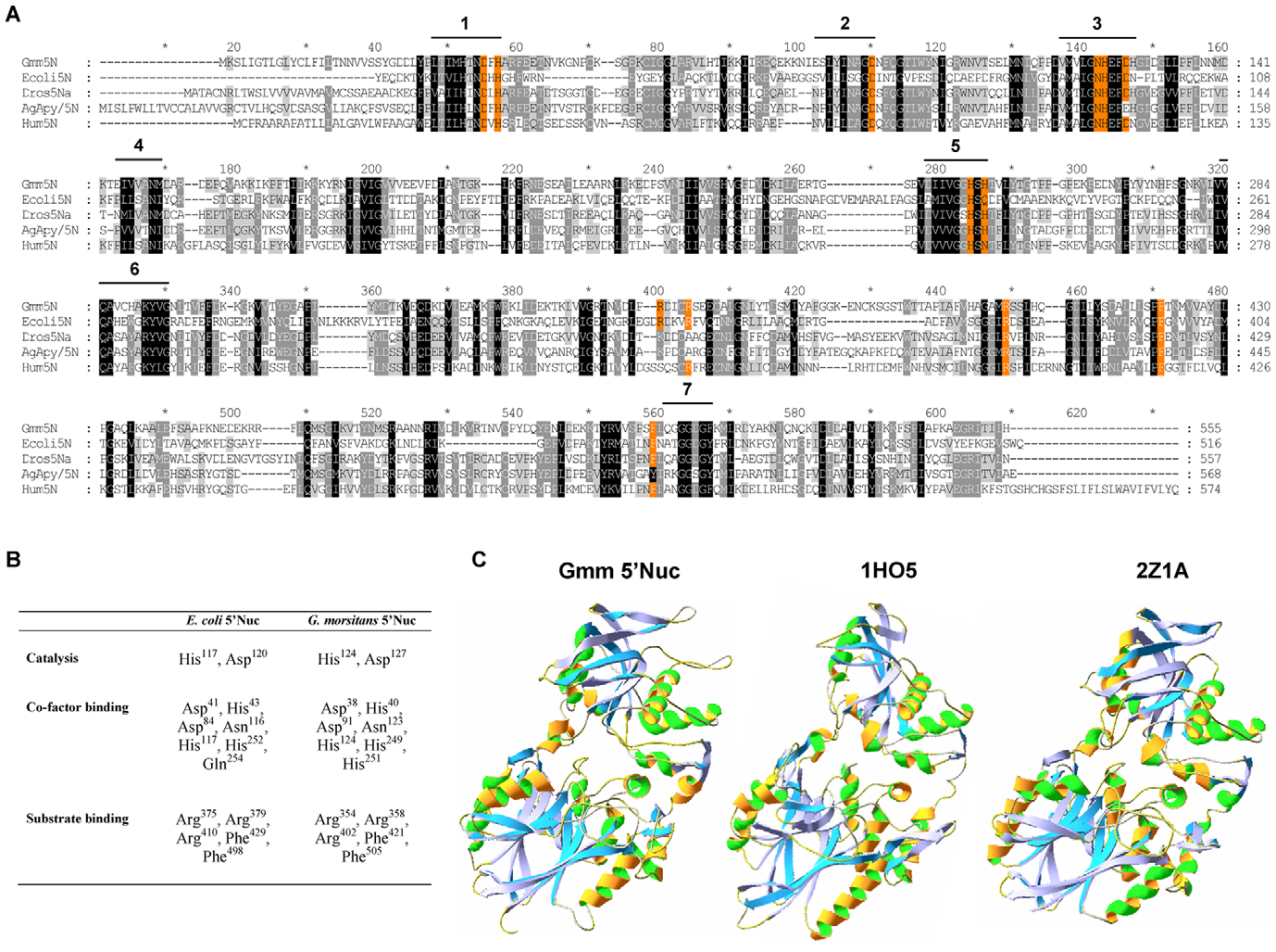
*In silico* analysis of the 5′Nuc cDNA. (**A**) Alignment of the *G. m. morsitans* 5′nucleotidase conceptual translation product with several members of the 5′nucleotidase family. Homologous residues are shown on a shaded background, residues that were identified for *E. coli* 5′Nuc to be important for catalysis, substrate and cofactor binding are indicated on an orange background. (**B**) Amino acids predicted to be involved in catalytic activity and co-factor and substrate binding of the *E. coli* 5′nucleotidase and their homologous residues in tsetse 5′Nuc (**C**). Structure prediction of tsetse 5′Nuc relying on comparison of Hidden Markov Models, based on resolved structures of homologous 5′nucleotidases. The predicted structure of tsetse 5′Nuc and the resolved structures of *Escherichia coli* and *Thermus thermophilus* 5′nucleotidases are depicted (respective PDBs: 1h05 and 2Z1a).

Screening of the λgt11 salivary gland cDNA library with the 385 bp 5′Nuc probe and subsequent sequencing of 5 positive clones identified a full-length 5′nucleotidase encoding cDNA of 1976 bp. (Fig. 1A). *In silico* analysis revealed a 102-bp 5′-untranslated region followed by an open reading frame corresponding to 555 amino acid residues and a 185-bp 3′-untranslated region that contains a polyadenylation signal (EMBL: AF384674).

Sequence analysis of the predicted translation product, designated as *G. m. morsitans* 5′nucleotidase-related protein (5′Nuc), identified the first 25 amino acids as a potential signal peptide leaving a mature protein of 530 amino acids with a calculated molecular weight of 59375 Da and an isoelectric point of 7.32. Four putative N-glycosylation sites (Asn^80^, Asn^173^, Asn^270^ and Asn^440^) and 2 O-glycosylation sites (Thr^256^ and Thr^389^) were identified. BLAST analysis revealed significant sequence similarities to a group of enzymes belonging to the 5′-nucleotidase family (Table S1) but lacks a hydrophobic C-terminal domain that signals for glycosylphosphatidylinositol anchoring. High degrees of similarity were apparent within the seven domains known to characterize enzymes exhibiting 5′nucleotidase activity [13] (Fig. 1A). Residues that were documented for *E. coli* 5′nucleotidase to be crucial for co-factor and substrate binding and catalytic activity [19], [20] are all present in the *G. m. morsitans* 5′nucleotidase-related protein (Fig. 1B). Structure modelling based on comparison of hidden Markov models, suggested strong structural similarity with resolved structures of the *Escherichia coli* and *Thermus thermophilus* 5′nucleotidase (respective PDBs: 1hO5 and 2Z1a, Fig. 1C).

### Identification of the 5′Nuc gene and expression analysis

**Figure 2:**
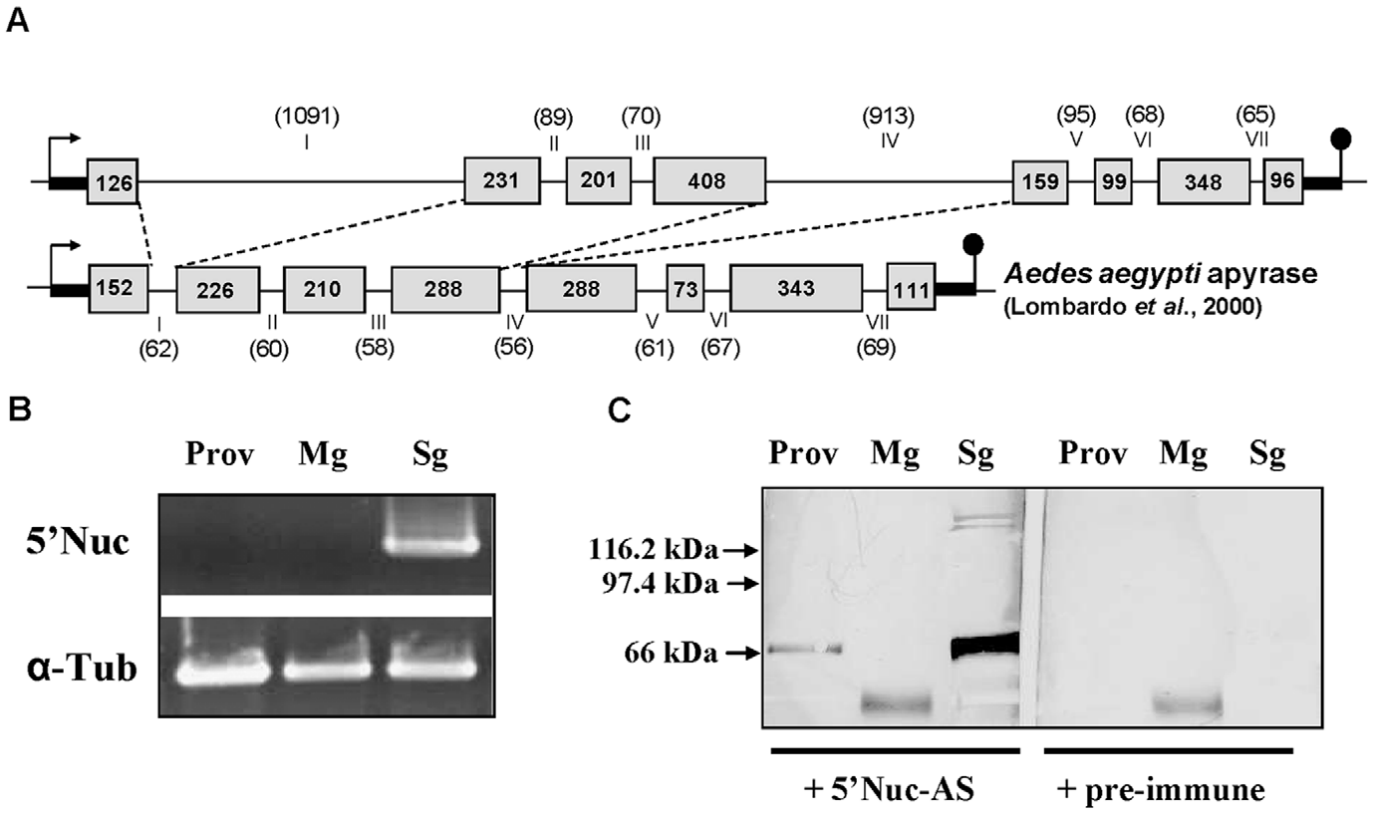
Identification of the 5′Nuc gene and its expression profile in tsetse flies. (**A**) Representation of the *G. m. morsitans* 5′Nuc gene as compared to the *Aedes aegypti* apyrase gene [12] with indication of intron and exon sizes, promoter and terminator regions. (**B**) RT-PCR based detection of 5′nucleotidase transcription in proventriculus, midgut and salivary gland tissue of 15-days-old male tsetse flies. The α-tubulin gene transcription was used as positive control. (**C**) Western blot analysis using rabbit anti-5′Nuc IgGs to evaluate the presence of the 5′Nuc protein in the proventriculus, midgut and salivary gland tissue of differently aged male tsetse flies.

The 5′Nuc structural gene of >4 kb including the 5′ and the 3′ UTRs was identified and revealed by Southern blot analysis to be present as a single copy in the tsetse fly genome. The gene was shown to contain 7 introns, including two with the size of approximately 1 kb (Fig. 2A). Northern blot analysis using salivary gland RNA corroborated that the gene resulted in a single transcript of around 1.7 kb, corresponding to the identified 1668 bp coding sequence (data not shown).

RT-PCR analysis revealed that 5′Nuc transcription occurs exclusively in the salivary glands (Fig 2B). Western blot analysis with anti-5′Nuc polyclonal rabbit serum resulted in a strong positive reaction around 65 kDa and a significantly weaker signal for the 125–140 kDa protein bands (previously identified as sgp3 that consists of a 5′nucleotidase domain, EMBL:EF398273, [21]) in the tsetse fly saliva (Fig. 2C). The molecular weight of 65 kDa as compared to the predicted 59.4 kDa suggested the presence of glycosyl modifications, as demonstrated using a glycosylation detection reagent and a mobility shift of about 5 kDa after PNGase F treatment (data not shown). In the soluble extract of the foregut/proventriculus tissue, a weak but significantly positive signal around 65 kDa could be detected (Fig. 2C), suggesting overflow of salivary proteins into this part of the alimentary tract. In teneral (newly emerged, non-fed) flies and at different time points after blood feeding, the relative abundance of the 65 kDa 5′Nuc protein in the total saliva protein pool remained at a similar constant level (data not shown).

### Total tsetse fly saliva exerts ATP(D)ase activity

**Figure 3:**
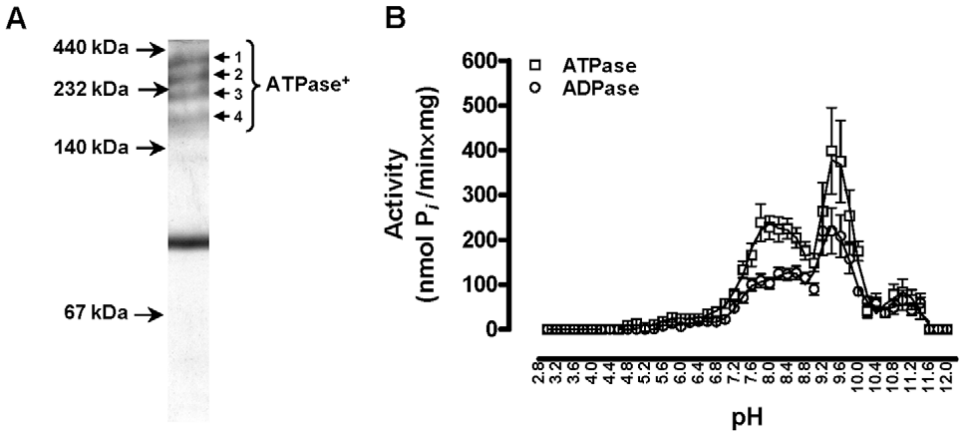
Apyrase activity in tsetse fly saliva. (**A**) Coomassie stained tsetse fly salivary proteins separated under native conditions. Protein bands 1 to 4 exerted ATPase activity as revealed by zymography. (**B**) pH dependence of the salivary ATP(D)ase activity, revealing two apparent alkaline pH optima (pH 7.8–8.6 and pH 9.4–9.6). No AMPase activity could be detected. Shown data are the compiled and representative results from five experiments using different saliva concentrations (1, 2.5 and 5 µg/ml).

Tsetse fly saliva exerts ATP(D)ase activity as illustrated in kinetic assays using ATP and ADP as substrates followed by determining the inorganic phosphate (P*_i_*) release. No AMPase activity could be detected, illustrating that tsetse salivary nucleotidases exert apyrase activity. Beside ATP and ADP, other nucleoside triphoshate and diphosphates substrates were converted such as rATP>rGTP = rUTP and rADP = rUDP>rCDP (data not shown). Separation of total tsetse saliva under native conditions followed by zymographic ATPase detection revealed activity confined to the upper four high molecular weight protein complexes (Fig. 3A) that are positive in a 5′Nuc-specific western blot (data not shown). Analysis of the pH dependence revealed alkaline pH optima around 8.0 and 9.5 (Fig. 3B). This salivary apyrase activity is dependent on divalent ions as cofactors with a preference for Mg^2+^ and Mn^2+^ and is completely inhibited by the addition of EDTA (data not shown).

### *In vivo* functional analysis of 5′Nuc by RNA interference (RNAi)

**Figure 4:**
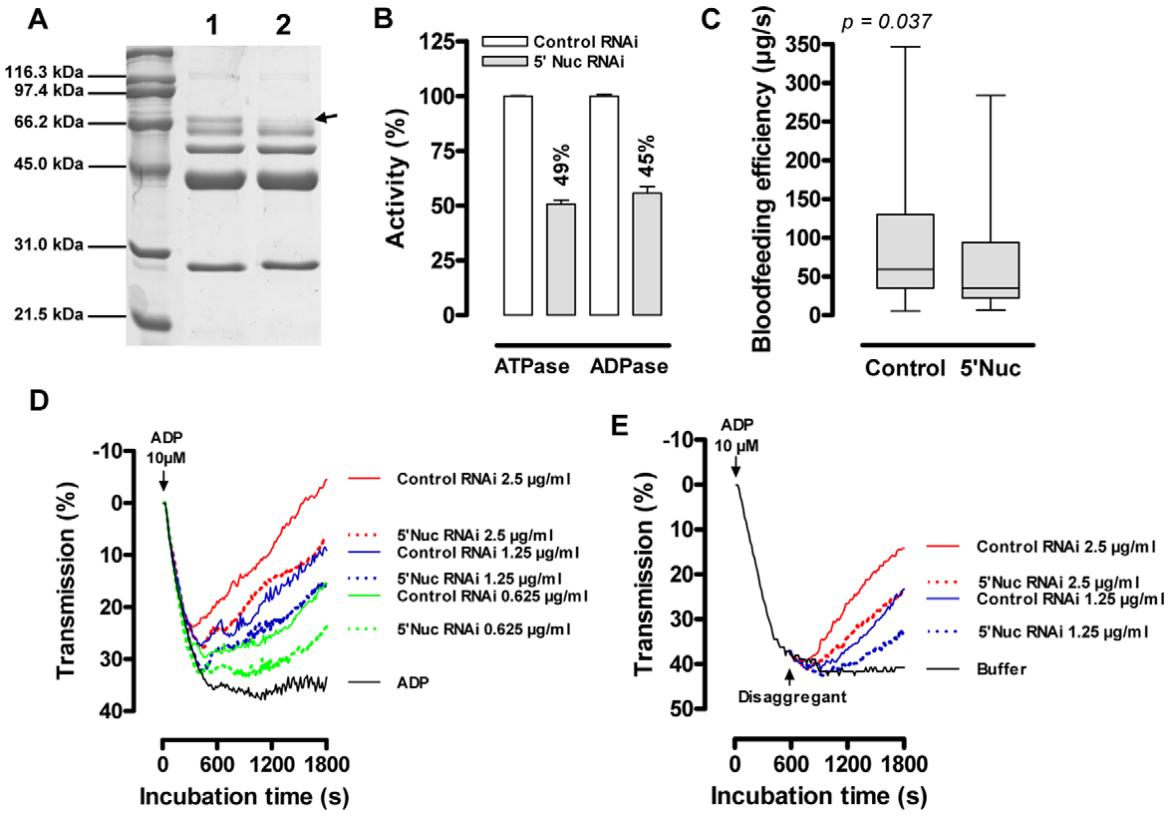
5′Nuc specific silencing by RNA interference. (**A**) Coomassie stained protein profiles of saliva harvested from control RNAi (lane 1) and 5′Nuc RNAi treated flies (lane 2) at day 12 post injection. The 5′Nuc protein band (confirmed by western blot analysis) is indicated with an arrow. (**B**) Inhibition (%) of respectively ATPase and ADPase activities by selective knockdown of 5′Nuc expression. (**C**) Blood feeding experiment monitoring the effect of silencing on feeding efficiency at day 12 after dsRNA injection. Influence of serially diluted saliva (2.5 – 0.625 µg/ml) from control (solid lines) and 5′Nuc RNAi treated flies (dotted lines) on (**D**) ADP-induced platelet aggregation and (**E**) disaggregation of maximally ADP-aggregated platelets. These results are representative of at least two independent experiments.

RNAi was applied for *in vivo* functional analysis of 5′Nuc by intrathoracal injection of 15 µg dsRNA per fly. This resulted in a highly specific knockdown of the 65 kDa 5′nucleotidase with 90% reduction at the mRNA level without silencing the homologous *sgp3* gene (Fig. S1). A maximal translational silencing of 75% was obtained at day 12 post injection as illustrated by densitometric analysis after SDS-PAGE (Fig. 4A). Saliva from these RNAi-treated and control dsRNA injected tsetse flies was compared in a P*_i_*-release based ATP(D)ase assay revealing an inhibition of the salivary ATPase activity by 49% and ADPase activity by 45% (Fig. 4B). As a result of silencing, feeding efficiencies on anesthetized mice of 5′Nuc RNAi flies (64±12 µg/s, *n* = 32) were significantly lower (*p* = 0.037, Fig. 4C) than those of control RNAi treated individuals (88±12 µg/s, *n* = 41). Indeed, flies with reduced salivary apyrase activity generally acquired smaller blood meals (13.0±1.5 mg versus 16.8±1.7 mg, *p* = 0.061) and required slightly longer times to complete blood feeding (267±28 s versus 248±27 s, *p* = 0.200). Evaluation of the inhibition of ADP-induced thrombocyte aggregation by ½ serially diluted saliva samples from RNAi treated flies (5 – 0.625 µg/ml, Fig. 4D), revealed an approximate 50% activity reduction upon 5′Nuc silencing. Also the capacity of saliva to disaggregate ADP-triggered platelets was reduced to a similar degree by 5′Nuc knockdown. To illustrate that the knockdown specifically affects the 5′Nuc activity, the potent inhibitory activity of tsetse saliva to bovine thrombin remained unaffected after 5′Nuc RNAi (data not shown).

### Refolding of recombinant 5′Nuc/SUMO fusion protein

**Figure 5:**
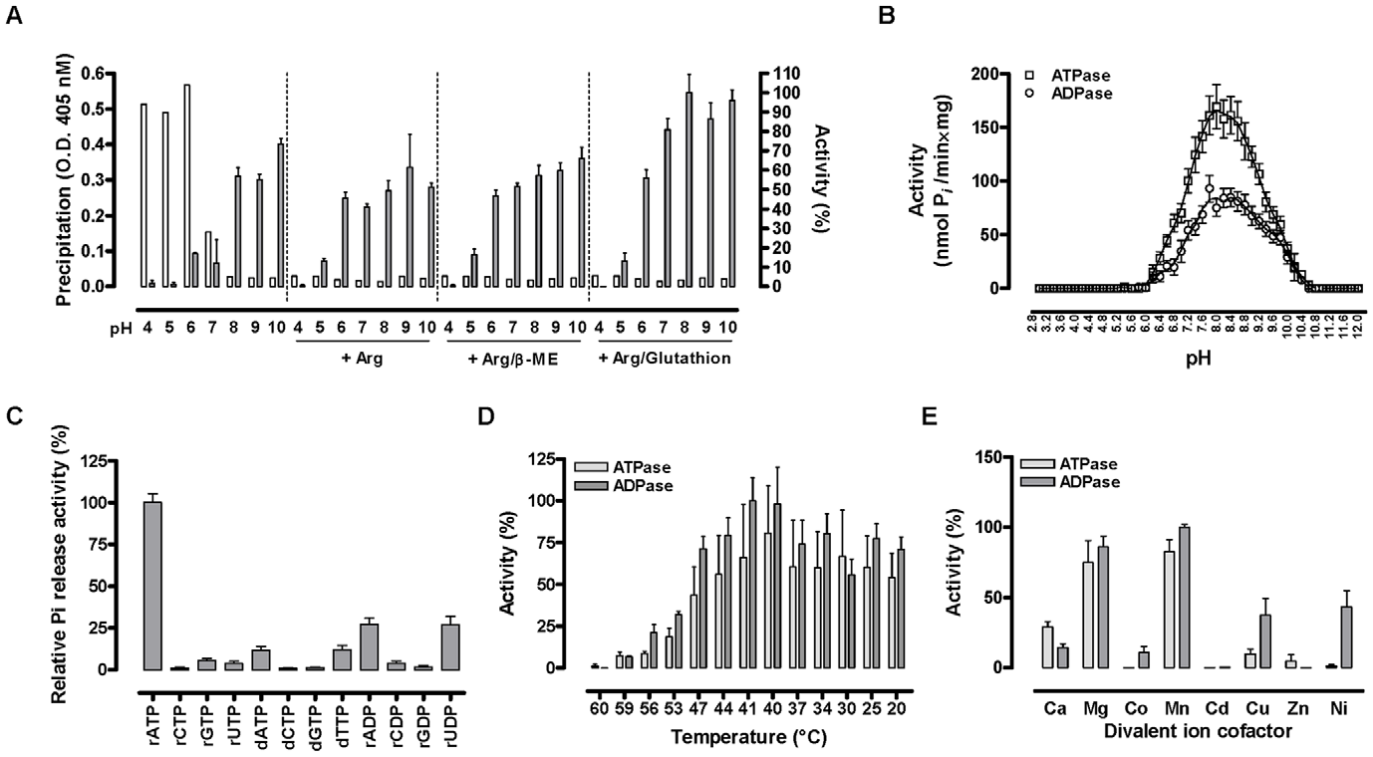
Enzymatic characterization of the 5′Nuc/SUMO fusion protein. (**A**) Refolding efficiency of 5′Nuc in different pH conditions (pH 4.0 to 10.0: 25 mM buffers of sodium acetate, MES, HEPES, Tris and piperazin supplemented with 250 mM NaCl, 5 mM KCl, 1 mM CaCl_2_ and 1 mM MgCl_2_) in the presence of additives such as 0.5 M L-arginine, 10 mM β-mercaptoethanol and an oxido-shuffling system (1 mM reduced and 0.1 mM oxidized glutathion). Primary read-outs for efficiency were precipitation (O.D. at 405 nm., empty bars, left Y-axis) and ATPase activity (% relative activity, filled bars, right Y-axis) determined using a malachite green based P*_i_*-release assay (**B**) pH dependence of the ATP(D)ase activity over a pH range from 3.0 to 12.0. Represented activities (nmol P*_i_*/min×mg) were calculated from the specific P*_i_*-release by 2.5 µg/ml recombinant protein from 20 µM ATP and ADP substrates in the respective buffers supplemented with 100 mM NaCl and 1 mM CaCl_2_ and 1 mM MgCl_2_ (**C**) Ribonucleoside and deoxyribonucleoside triphosphates and ribonucleoside diphosphate specificities were determined in a kinetic assay using 20 µM of the individual substrates in the presence of 1mM Mg^2+^ and 1mM Ca^2+^ at pH 8.0. Activities were calculated percentual to the rATPase activity. (**D**) Temperature dependence of recombinant 5′Nuc in an interval between 20 and 60°C. Activities (%) were calculated as relative to those measured in optimal temperature conditions. (**E**) Percentage ATPase and ADPase activity in the presence of different co-factors (Ca^2+^ Mg^2+^, Co^2+^, Mn^2+^, Cd^2+^, Cu^2+^, Zn^2+^ and Ni^2+^) was measured in the presence of 0.1 mM EDTA and EGTA. The enzyme was pre-incubated for 15 minutes with the different co-factors prior to addition of 20 µM substrate. Activity was measured over 60 minutes and calculated within the linear phase of substrate conversion. All data are representative of at least two independent experiments.

Recombinant 5′Nuc/SUMO fusion protein, purified from inclusion bodies using immobilized metal affinity chromatography, was refolded by rapid dilution in different non-denaturing buffers. Various pH conditions (pH 4.0 to 10) in combination with additives such as 0.5 M L-arginine, 10 mM β-mercaptoethanol and oxidized/reduced glutathion (0.1 mM/1 mM) were tested. Primary read-out for the resolubilisation and refolding efficiency were precipitation (O.D. 405nm) and ATPase activity (Fig. 5A). Recombinant 5′Nuc could be readily solubilized in alkaline buffers from pH 8 onwards without the addition of L-arginine. Gain-of-activity was obtained without the requirement of an oxido-shuffling system (Fig. 5A). Moreover, reducing agents such as β-mercaptoethanol and dithiothreitol did not affect ATPase activity, illustrating that disulfide bridges are not required for activity of 5′Nuc.

### Enzymatic characteristics of refolded 5′Nuc/SUMO fusion protein

**Figure 6:**
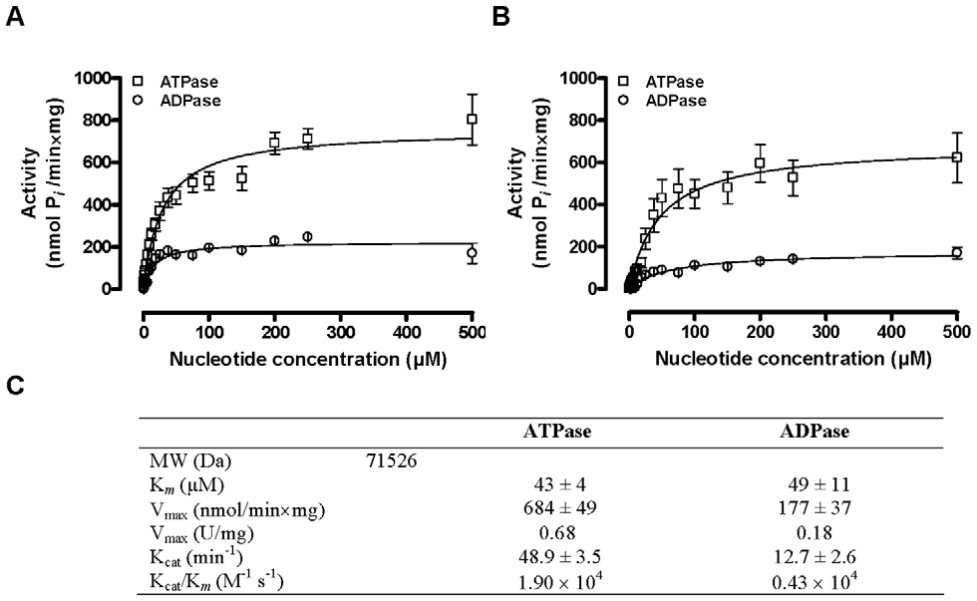
Determination of 5′Nuc biochemical parameters. Michaelis Menten plot illustrating the P*_i_* release by (**A**) 1 µg/ml total tsetse fly saliva and (**B**) 2.5 µg/ml 5′Nuc from increasing concentrations of ATP and ADP substrate (0.78 to 500 µM) in a 200 µl reaction volume in the presence of 1 mM Mg^2+^ and Ca^2+^. (**C**) Kinetics values (Km, Vmax, Kcat and Kcat/Km) for recombinant 5′Nuc/SUMO fusion protein, determined based on three experiments.

Refolded monomeric 5′Nuc exerted ATPase and ADPase but no AMPase activity, similar to what was observed for total tsetse fly saliva. Evaluation of the pH dependence illustrated a preference for alkaline conditions, with an optimum at pH 8.0 for both ATPase and ADPase activity (Fig. 5B). Different nucleoside triphosphate and diphoshate substrates could be converted such as rATP≫dATP = dTTP>rGTP = rUTP and rADP = rUDP>rCDP (Fig. 5C). The temperature optimum for the ATP(D)ase activity was around 40°C but remained high in a broad range of suboptimal temperature conditions (20°–47°C) (Fig. 5D). Similar as for total saliva, ATPase and ADPase activities of the recombinant 5′Nuc depended on divalent ions with a preference for Mn^2+^ and Mg^2+^. To a lesser extent, Ca^2+^ could also activate ATPase and ADPase activity, while Cu^2+^, Ni^2+^ and Co^2+^ support mainly ADPase activity (Fig. 5E). ATPase and ADPase experiments that were performed with total tsetse fly saliva (Fig. 6A) and refolded 5′Nuc (Fig. 6B) suggested that the native and recombinant enzymes obeyed Michaelis-Menten kinetics, allowing the estimation of K_m_ and V_max_ from non-linear regression analyses. K_m_ values were determined in three independent experiments, being 43±4 µM for ATPase and 49±11 µM for ADPase activity of recombinant 5′Nuc. V_max_ values were determined to be 684±49 and 177±37 nmol P*i*/min×mg for respectively ATPase and ADPase activities (Fig. 6C). Comparable K_m_ (33±4 µM for ATPase and 19±3 µM for ADPase) and V_max_ values (791±27 and 239±11 nmol P*i*/min×mg respectively) were obtained for total tsetse fly saliva. K_cat_/K_m_ ratios confirmed a substrate preference of 5′Nuc for ATP over ADP (Fig. 6C).

Next, a range of ATPase/nucleotidase inhibitors was tested for their influence on the ATP(D)ase activity of 5′Nuc. While 1 mM of the Na^+^/K^+^-ATPase inhibitor ouabain and alkaline phosphatase inhibitor levamisole had no influence on 5′Nuc, 1 mM adenosine, AMP and AP5A as well as 1 mM vanadate and 10 mM NaN_3_ had moderate inhibitory effects on enzymatic activity (Table S2). DEPC (2 mM), sodium fluoride (10 mM) and 4,4′ diisothiocyanostylbene 2,2′ disulfonic acid (DIDS, 100 µM) nearly completely abrogated substrate conversion while concanavalin A enhanced the activity as has been described for other 5′ nucleotidases (Table S2).

### Fibrinogen receptor (GPIIb/IIIa) binding properties of refolded 5′Nuc/SUMO fusion protein

**Figure 7:**
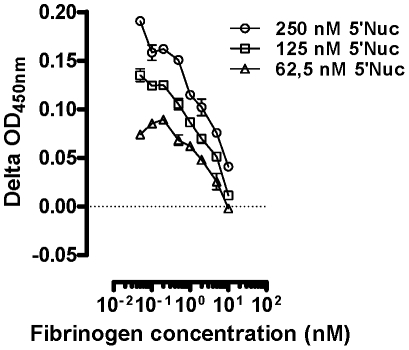
5′Nuc binding onto purified human fibrinogen receptor (GPIIb/IIIa). 5′Nuc binding (250, 125 and 62.5 nM) onto solid-phase immobilized GIIb/IIIa in the presence of varying concentrations of fibrinogen (20 – 0.05 nM), detected using a rabbit anti-5′Nuc polyclonal IgG. These results are representative of two independent experiments.

To evaluate the fibrinogen receptor antagonistic properties of 5′Nuc/SUMO, its binding to solid-phase immobilized GIIb/IIIa was evaluated. Using both a SUMO-Tag specific and an anti-5′Nuc IgG based detection, concentration-dependent 5′Nuc binding onto the purified receptor could be demonstrated. Based on four experiments, an apparent K_D_ of 92±25 nM was calculated. To further illustrate the specificity of the binding, inhibition studies were performed using varying concentrations of purified human fibrinogen (Fig. 7). These illustrated a dose-dependent inhibition of 5′Nuc binding by fibrinogen, further confirming that the tsetse fly 5′Nuc has GIIb/IIIa receptor-specific antagonistic properties.

### Platelet inhibitory properties of refolded 5′Nuc/SUMO fusion protein

**Figure 8:**
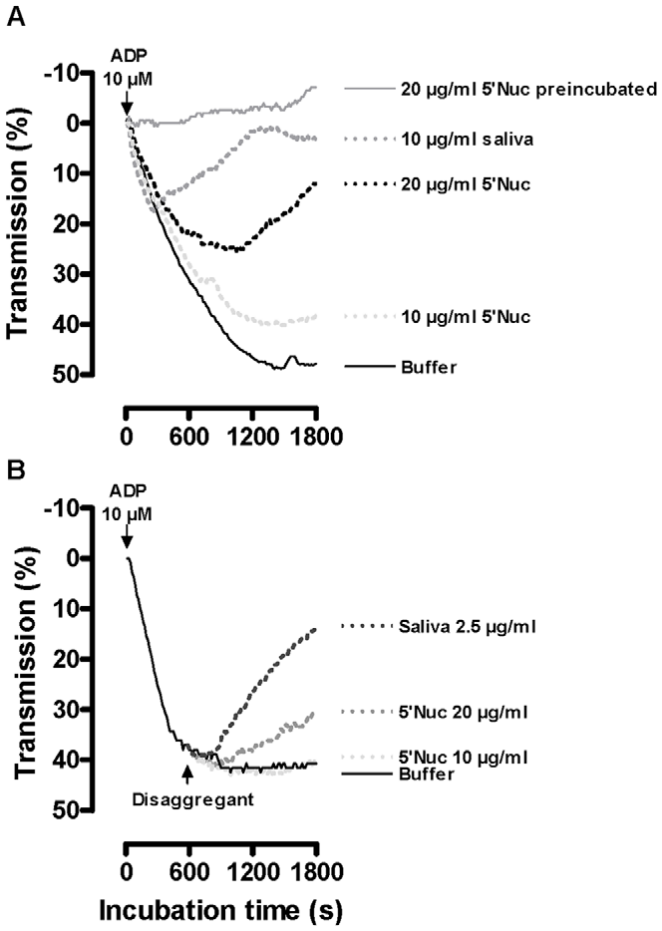
5′Nuc thromboregulatory activity. (**A**) Inhibitory action of 5′Nuc (10 and 20 µg/ml, simultaneous or preincubated with the 10 µM ADP) and saliva (10 µg/ml) on ADP-induced platelet aggregation. (**B**) Disaggregating potential of saliva and 5′Nuc on ADP-aggregated thrombocytes. Addition of ADP and disaggregants are indicated with arrows. These results are representative of at least two independent experiments.

Recombinant 5′Nuc/SUMO protein was assessed for its activity on human platelets using an aggregation assay in microtiter plates. Similar as for total tsetse fly saliva, the recombinant protein inhibited ADP-induced platelet aggregation and even caused disaggregation of thrombocytes in a dose-dependent manner (Fig. 8A). Pre-incubation of ADP with the recombinant protein completely abrogated the ADP-induced platelet response (Fig. 8A). Exposure of maximally ADP-aggregated platelets to saliva or recombinant 5′Nuc resulted in a disaggregation response that for 5′Nuc was significant at the highest tested concentration of 20 µg/ml (Fig. 8B).

## Discussion

Tsetse fly saliva is a complex mixture of proteins that is essential for the blood feeding process which requires the creation of a blood pool at the bite site and the maintenance of blood fluidity in the mouthparts, crop and anterior midgut. Previously, it was shown that an ADP-hydrolyzing activity, present in a crude salivary extract, can inhibit platelet aggregation (Mant & Parker, 1981).

In this report, we functionally characterized a secreted 65 kDa 5′nucleotidase-related protein (5′Nuc, EMBL: AF384674) as a major apyrase and fibrinogen receptor (GPIIb/IIIa) antagonist that is estimated to make up about 5% of the tsetse fly saliva protein pool [21]. Experimental evidence is based on the successful suppression of 5′Nuc transcription in the tsetse salivary gland by RNA interference and on the production of functional recombinant protein with characteristics that are nearly identical to the native activity. This recombinant 5′Nuc still had a strong tendency to aggregate in inclusion bodies despite the presence of a SUMO-chaperone for better folding [22]. However, the advantage of purifying 5′Nuc from these inclusion bodies was the possibility to obtain homogeneous preparations, as problems of proteolytic degradation and copurification with contaminants has precluded biochemical characterisation of several orthologs. Out of the different refolding conditions that were tested, it was sufficient to make a fast dilution of the protein into a non-denaturing alkaline buffer with pH>8.0, without the need of folding-assisting agents to obtain a good yield of active protein. Successful refolding in similar alkaline conditions has also been reported for rat NTPase ectodomains [23]. The active monomeric 5′Nuc that we obtained by size exclusion chromatography after refolding was clearly an apyrase as it excluded AMP as substrate. Preferred substrates were rATP, rADP and rUDP while other nucleoside tri- and diphosphates were less potently converted. The enzyme had also a preference for ribonucleoside over deoxyribonucleoside triphosphates as rATP was a much better substrate than dATP. These base and sugar preferences of 5′Nuc provide the enzyme with the most relevant nucleotide specificities (for rATP and rADP as platelet triggers) in the context of anti-hemostasis. Co-factor studies using recombinant protein and total tsetse fly saliva identified Mn^2+^ and Mg^2+^ as most effective, while Ca^2+^ could only induce moderate activity. This is similar to the triatomine 5′nucleotidase related apyrase [15], while most other identified apyrases of blood feeding insects have a cofactor preference for Ca^2+^ [10], [14], [24]. Cu^2+^ and Ni^2+^ were also found to activate the enzyme (primarily stimulating ADPase activity), similar as for some *E. coli* nucleotidases [25], whereas Cd^2+^ and Zn^2+^ could not induce the tsetse fly 5′Nuc although these are suitable cofactors for the *E. coli* homologue [19]. Assumptions on the mechanism of substrate dephosphorylation as well as a prediction of the tsetse 5′Nuc structure could be made based on previous studies on the homologous *E. coli* 5′nucleotidase [19], [20]. Structure prediction suggested a protein fold consisting of two domains, linked through an α-helix (see Fig. 1C). The *E. coli* homologue has the ability to shift between an open (inactive) and closed (active) conformation as a result from an interdomain rotation of 96°. At the interphase of the two domains, at the C-terminal end of 2 sandwiched βαβαβ-motifs, the dimetal catalytic site is located. All residues that were illustrated to coordinate the metal ions in *E. coli* 5′Nuc are also present in the homologous *Glossina m. morsitans* 5′Nuc (Asp^38^, His^40^, Asp^91^, Asn^123^, His^124^, His^249^). Only the *E. coli* Gln^254^ is substituted by a histidine (His^251^), a replacement which is also found in e.g. human, *Drosophila* and *Anopheles gambia*e 5′Nuc. The Asp-His dyad that is part of the catalytic core structure as well as most of the residues that constitute the substrate binding pocket are conserved in tsetse 5′Nuc (respectively His^124^ and Asp^127^ and Arg^354^, Arg^358^, Arg^402^, Phe^421^ and Phe^505^). The crucial involvement of the bivalent cations and the histidine that might function as a general base to drive the nucleophilic attack by one of the coordinated water molecules was illustrated by the efficient inhibition by respectively EDTA and DEPC. Disulfide bridges and glycosylation were suggested to have little influence on the core structure and enzymatic function of other 5′nucleotidase because the involved cystein residues are already in close proximity and glycosylated asparagine residues are located at the surface of the molecule [26]. Our data indeed show that reducing agents (β-mercaptoethanol and DTT) do not affect catalytic rates and that the non-glycosylated recombinant protein (expressed in *E. coli*) exerts potent activity. The fact that AMP does not serve as a substrate might suggest that AMP is not properly accommodated in the active site and that the α-phosphate group cannot be hydrolytically removed by the tsetse 5′Nuc. Given that AMP as well as adenosine and AP5A had some inhibitory effects (20–50%) on 5′nucleotidase activity at a 50× molar excess, these adenosine derivatives probably bind to the active site with low affinity, where the Phe^429^ and Phe^498^ residues could be responsible for stacking of the adenine ring [20]. Nevertheless, the functional result is that the saliva converts ATP and ADP released at the bite site not further than AMP, where AMP has been suggested to promote the blood feeding process by inducing vasodilatation [27]. As adenosine is not produced, the functional relevance of two tsetse fly salivary proteins with putative adenosine deaminase activity, tsetse salivary gland growth factors 1 and 2 (TSGF-1 and TSGF-2) [28], remains unclear.

Biochemical characterisation of the refolded recombinant 5′Nuc/SUMO protein revealed K_m_ values that were in the low micromolar range (43 µM and 49 µM) and maximal enzymatic rates of 684±49 and 177±37 nmol P*_i_*/min×mg or 0.68 and 0.18 U/mg for respectively ATPase and ADPase. Although limited biochemical information is available on apyrases of blood feeding arthropods, the measured K_m_ values were comparable to those described for e.g. a recombinant mosquito (*Aedes aegypti*) apyrase [29] and the porcine hepatic canalicular ATP(D)ase [30]. With the definition of 1 unit corresponding to the amount of enzyme required to release 1 µmol of P*_i_*/min and the fact that tsetse flies inoculate approximately 4 µg saliva at the feeding site to obtain a 20 µl blood meal, this would correspond to 100 mU/ml ATPase and 30 mU/ml ADPase under V_max_ conditions (0.79 and 0.24 U/mg for saliva). This inoculated activity, which is confined to 4 high molecular weight (HMW) protein complexes as revealed by native gel separation and zymography, would theoretically be sufficient to convert local concentrations of 200 µM ATP and 70 µM ADP within an average feeding time of 4 minutes. The physiological advantage of these HMW complexes could be the centralization and potentation of several pharmacological activities similar as what has been described for snake venom (reviewed in [31]) and a reduction in diffusion rate from the blood pool where the tsetse fly feeds.

The exclusive expression of 5′Nuc in the salivary gland tissue suggested that the activity is primarily required in the saliva inoculum at the bite site. In this context, the potency of tsetse saliva to counter ADP-induced hemostasis has been previously documented [4] and our thrombocyte aggregation assays have illustrated the potency of 5′Nuc to inhibit and even reverse ADP-induced platelet responses, a feature which was also described for a 5′nucleotidase related fibrinogen receptor (GPIIb/IIIa) antagonist in the saliva of *Chrysops* deerflies [32]. 5′Nuc/SUMO binding studies onto purified human GPIIb/IIIa combined with fibrinogen inhibition studies have clearly illustrated that the tsetse fly 5′Nuc is also a fibrinogen receptor antagonist, where the recombinant protein displayed an apparent K_D_ of 92±25 nM. This feature is likely causally linked to the platelet disaggregating potential of 5′Nuc in tsetse fly saliva. The binding mode of 5′Nuc and the homologous chrysoptin to GPIIb/IIIa remains to be elucidated, as both lack the characteristic RGD-motif that has been recurrently found in other fibrinogen receptor antagonists [32]. Moreover, these data illustrate that, unlike chrysoptin, the glycosylation is not absolutely required for GPIIb/IIIa binding of the tsetse fly 5′Nuc.

In addition to influencing ADP-induced hemostasis, mediated through P2Y_1_ and P2Y_12_ receptor ligation and involving downstream fibrinogen-mediated reactions, substrate preference of 5′Nuc for ATP suggested that interference with ATP-based host reactions plays an important role in the blood feeding process. It is known that ATP can bind to the P2X_1_ receptor on platelets thereby amplifying their responsiveness to various activating stimuli. This feature has been illustrated to especially contribute to hemostasis in high shear rate conditions that might occur in the microvasculature at the blood feeding site [33]. Beside the involvement in thromboregulation, extracellular ATP was also shown to induce IL-1β and IL-18 and to stimulate phagosome-lysosome fusion in macrophages [34], [35], to provoke chemotaxis and distorted maturation of dendritic cells [36], [37] and to amplify the chemotactic gradient that orients granulocyte migration [38]. In line with these properties of ATP and ADP, the presence of ectonucleotidase activities on the surface of various cell types has been shown to be important in thromboregulation [39] and the control of inflammatory reactions in the host [40], [41]. As such, salivary apyrases of hematophagous arthropods might not only inhibit several aspects of hemostasis but also reduce inflammation at the bite site. Possibly, the documented anti-inflammatory properties of nucleotidases explain the salivary immunomodulatory action that we have previously described to promote trypanosome infection onset [18].

In addition to its activity at the blood feeding site, analysis of the apyrase activity in total saliva revealed that optimal activities were obtained in alkaline pH conditions that corresponded to the measured pHs in respectively saliva (pH 7.8) [21] and the proventricular tract (pH 9.5). Together with the observation that 5′Nuc leaks into the proventriculus, this suggested that the activity is not restricted to the salivary gland and micro-environment of the bite site but that it might also act in the upper part of the alimentary tract. Our *in vivo* gene silencing experiments revealed that 5′Nuc supports the blood feeding process as evidenced by slower feeding rates for RNAi-treated flies that generally obtained smaller blood meals and required slightly longer times to feed. This blood feeding phenotype resulted from a 50% knockdown of apyrase and anti-thrombotic activity despite the remaining 25% 5′Nuc protein and the presence of another putative apyrase in tsetse fly saliva, the sgp3 protein that we have previously identified [21] and that remained unaffected by the RNAi. Detailed analysis of the salivary gland EST database ([42], http://www.genedb.org/genedb/glossina/) further supports the strong dominance of the *5′nuc* that is represented by 25 ESTs in a total of 37 EST clusters putatively encoding 5′nucleotidase-related proteins. Another cluster of 10 ESTs encodes Sgp3 that still remains to be characterized as a true apyrase, while another candidate, GM-544, is only represented by 2 ESTs that are moreover truncated at the 3′ end. Collectively, based on the *in vivo* silencing, the biochemical analyses and platelet aggregation assays, we have calculated that the 65 kDa 5′Nuc accounts for approximately 65% of the total ATP/ADP-hydrolyzing activity and the bulk of the anti-platelet aggregation/disaggregating activity in tsetse saliva and have uncovered its fibrinogen receptor antagonistic potential. These data clearly illustrate the important anti-hemostatic role of 5′Nuc in the tsetse feeding biology and elucidation of its exact mode of binding to the fibrinogen receptor might be of significant biomedical interest.

## Materials and Methods

### Ethics statement

Blood sampling from 3 human volunteers was performed after obtaining a written informed consent. These individuals are the authors and co-author of this manuscript (GC, JVDA and KDR) that have been informed on the purpose of the sample collection that was approved by the Ethics Committee of the Institute of Tropical Medicine (ITM), Antwerp (Belgium).

Animal ethics approval for the tsetse fly feeding on mice and rabbits was also obtained from the Institutional Review Board of ITM.

### Chemicals

Nucleoside diphosphates and monophosphates and inhibitors [4,4′-diisothiocyanostylbene 2,2′-disulfonic acid (DIDS), ouabain, sodium orthovanadate, sodium azide, sodium fluoride, DEPC, levamisole, P^1^P^5^-di(adenosine-5′)pentaphosphate (AP5A), concanavalin A and adenosine] were purchased from Sigma. Ribonucleoside and desoxyribonucleoside tri- and diphosphates were purchased from Roche.

### Tsetse flies, tissue collection and saliva harvesting

Tsetse flies (*Glossina m. morsitans*, ITMA colony) were available from the insectaria at the Institute of Tropical Medicine Antwerp. Adult male flies were starved for 72 hours prior to dissection (at day 15 after emergence) of three different tissues: (i) the entire midgut (posterior + anterior), (ii) the proventriculus and foregut and (iii) the salivary glands. The tissues of 30 individuals were pooled into microtubes containing 60 µl sterile H_2_O and stored at −20°C until analysis. Saliva was harvested from the salivary gland tissue as described earlier [43], protein concentrations were determined by the BCA kit (Pierce Biotechnology) and aliquots stored at −20°C.

### SDS-PAGE electrophoresis and protein electrotransfer

Tissue samples were thawed, homogenised with a Teflon pestle and run under reducing and denaturing conditions on 10% SDS PAGE. Proteins were stained with Coomassie Brilliant blue or by silversalts, or electrotransferred to nitrocellulose (Hybond C, Amersham) or PVDF membranes. Some protein bands were subjected to densitometric analyses as described earlier [44]. Detection of glycosyl modifications was achieved with the Gelcode glycoprotein staining kit (Pierce) and mobility shift assays were performed using native and PNGase F treated saliva.

### N-terminal amino acid sequencing

The N-terminal amino acid sequences of selected salivary protein bands were determined by automated Edman degradation in an ABI 471-B sequencer, operated as recommended by the manufacturer.

### cDNA library screening and clone isolation

A 385 base pair fragment, corresponding with the 5′ part of the 5′nucleotidase cDNA, was amplified using two primers that target respectively the 5′ untranslated region (sense primer: 5′-CCTAAATCCTTTCTTTAAC-3′) and the region encoding the amino-terminal part of the protein (antisense primer: 5′–CACGTGCCAGACCACCGATGC-3′). To be used as a DNA probe for screening of the λgt11 salivary gland cDNA library, the PCR product was purified and labelled by random priming with dUTP-digoxigenin using the DIG High Prime DNA Labeling and Detection Starter Kit II (Roche). Approximately 20000 plaques were lifted with Nylon membrane (Roche) and hybridized with this specific probe. Five positive plaques were picked, confirmed by PCR and screened an additional two rounds with the same probe. Isolated positive plaques were amplified in the *E.coli* Y-1090 strain and phage DNA was purified using the Wizard Lambda Preps DNA Purification System (Promega).

### cDNA sequence analysis and protein structure prediction

The obtained full-length cDNA was excised from the λgt11 arms with the *Sfi* and *Not*I restiction enzymes (Roche), ligated into pGEM-13Zf(+) vector (Promega) and transformed into Top 10 *E. coli* cells. Clones were sequenced using T7 and Sp6 primers as well as custom primers constructed from the internal sequence of the cDNA clone. Translated sequences were aligned using the CLUSTALW program and imported into GeneDoc (www.psc.edu/biomed/genedoc). Signal peptide prediction was based on the SPScan program (GCG software package). Homology detection and structure prediction was based on comparison of Hidden Markov Models (HHpred, http://toolkit.tuebingen.mpg.de/hhpred). Generated PDB-files were used to produce 3D-figures using Deepview/Swiss-PdbViewer (http://www.expasy.org/spdbv/).

### 5′Nuc gene identification

Using *G. m. morsitans* salivary gland genomic DNA as a template, a PCR was performed using a sense primer in the 5′UTR (5′-CCTAAATCCTTTCTTTAAC-3′) and a 3′end primer (5′-ATGAATAATCGTAATGCG-3′) that span the entire coding sequence. The >4 kb amplification product was sequenced by using an additional number of internal primers. A universal GenomeWalker Kit (Clontech) was used to determine the 5′ and 3′ flanking sequences. A putative promotor sequence and the splicing sites were identified using software analysis tools (Neural Network Promotor prediction and Splice Site Prediction by Neural Network) that are available at the Berkeley *Drosophila* Genome project website (http://www.fruitfly.org/). The predicted splicing sites were confirmed by comparison with the cDNA sequence.

### 5′Nuc cloning and recombinant expression in *E. coli*

The 5′Nuc encoding cDNA was PCR-amplified without signal sequence from the λgt11 *Sfi*-*Not*I fragment in pGEM13f(+) using the GmSG4 (5′-TTTTGGTCTCTAGGTGACGATTTATATCCTC-3′) and GmSG5 (5′-TTTTGGATCCTTAATGAATAATCGTAATGC–3′) primers. The amplification product was unidirectional cloned into pSUMO (LifeSensors) using *Bsa*I and *BamH*I restriction sites. The expression plasmid was transformed in BL21(DE3) *E. coli* cells and expression was induced by 1 mM isopropyl-1-thio-β-galactopyranoside (IPTG) for 3 hours at 20°C. Next, cells were harvested, resuspended in lysis buffer [50 mM Tris pH 8.0, 600 mM NaCl, Complete protease-inhibitor (Roche)] and lysed by freeze-thawing and sonication. Inclusion bodies were rinsed twice with 1% Triton-X-100 and resolubilized in 6 M guanidinium hydrochloride in the same buffer conditions.

### Recombinant 5′nucleotidase purification, refolding and antibody production

The SUMO-tagged 5′Nuc was purified on Ni-NTA (Sigma), eluted in 6 M urea with 0.5 M imidazole and refolded in pH conditions ranging from 4.0 to 10.0 [sodiumacetate (pH 4.0 to 5.4), MES (pH 5.6 to 6.6), HEPES (pH 6.8 to 7.8), Tris (pH 8.0 to 9.0) and piperazin (pH 9.0 to 10.0)] in the presence and absence of additives such as 0.5 M arginine, 1 mM reduced and 0.1 mM oxidized glutathion and 10 mM β-mercaptoethanol. Refolding efficiency was determined by quantifying precipitation (O.D. 405 nM) and ATPase activity. For the biochemical characterisation experiments, recombinant 5′Nuc was refolded in 25 mM piperazin pH 10.0 supplemented with 250 mM NaCl, 5 mM KCl, 2 mM CaCl_2_ and 2 mM MgCl_2_. Refolding was obtained by a fast injection and 1/20 dilution of the 5′Nuc Ni-NTA elutes (at approximately 1 mg/ml) into the refolding buffer and incubation for 3 to 7 days at 4°C on a rocking platform at 70 rpm. Next, the 5′Nuc protein was concentrated using centrifuge concentrators with polyethersulfone membranes and a 50 kDa molecular weight cut-off. Concentrates were run on a Superdex 200 gelfiltration column connected to an Äkta explorer (GE Healtcare), allowing change of buffer to 1 mM piperazin pH 9.5, 100 mM NaCl and separation of active monomeric 5′Nuc from aggregates and impurities.

5′Nuc was also expressed with a T7-tag, affinity purified on a T7-tag antibody agarose column (Novagen) and used for raising rabbit polyclonal antiserum for Western blot analyses.

### Southern, Northern and Western blot analyses and RT-(q)PCR

A P^32^ labelled probe was prepared by PCR, using primers encompassing the entire 5′nucleotidase coding sequence (1668 bp). The same probe was used for both Southern and Northern blot analysis. Northern blot was performed on salivary gland RNA from adult male flies. Southern blot analysis was performed on genomic DNA, from *G. m. morsitans* mixed males and females, digested by a range of restriction enzymes (*Pst*I, *Xho*I, *Bam*HI, *Ban*II, *Sac*I, *Eco*RI and *Hind*III). Western blot was used to assess expression in different tissues using the raised rabbit anti-5′Nuc polyclonal serum (1/1000) and a peroxidase-conjugated anti-rabbit IgG (1/1000, Sigma). Conventional and quantitative RT-PCR analyses were performed using following primers: 5′Nuc sense (5′-CGGGTAATAAAGTTCTGGTCGTA-3′), 5′Nuc antisense (5′-TTGGCAAGTCCACATTTGTTCTC-3′) and primers that were used in a previous study [44]. The PCR cycles (35 for the conventional PCR, 45 for the qPCR) consisted of 1 min. denaturation at 94°C, 45 s. annealing at 54°C and 1 min. extension at 72°C. Gene expression was normalized using tubulin, actin and TAg5.

### Nucleotidase activity measurements by detection of P*_i_* release

Zymographic detection of ATPase activity in total saliva was performed by separating the proteins at 4°C in native conditions on an 8% polyacrylamide gel using TAE (40 mM Tris, 5 mM sodium acetate, 1 mM EDTA) as running buffer and applying a voltage of 100V for 3 hours. ATPase activity was revealed by zymography as described elsewhere [10].

Nucleotidase activities of total tsetse fly saliva and refolded recombinant 5′Nuc were measured by quantification of the P*_i_* release from the individual substrates (rATP, rCTP, rGTP, rUTP, dATP, dCTP, dGTP, dTTP, rADP, rCDP, rGDP and rUDP). Saliva (1–5 µg/ml) and recombinant 5′Nuc/SUMO (2.5–5 µg/ml) mediated activity on the substrates (20 µM) in a 200 µl reaction volume was analysed using the Malachite Green phosphate assay kit (Gentaur) and O.D. measurement (λ = 630nm) in an Ultra microplate reader ELX808 (Bio-Tek Instruments).

For pH dependence analyses, reactions were performed in 25 mM buffers (with 100 mM NaCl) ranging from 3.0 till 12.0 with pH differences of 0.2. The used buffers were sodiumacetate (pH 3.0 to 5.4), MES (pH 5.6 to 6.6), HEPES (pH 6.8 to 7.8), Tris (pH 8.0 to 9.0) and piperazin (pH 9.0 to 12.0). Reactions were performed in the presence of 1 mM of CaCl_2_ and MgCl_2_. To determine the cofactor preference, activity was monitored in the presence of a range of divalent ions (Ca^2+^, Mg^2+^, Co^2+^, Cd^2+^, Cu^2+^, Zn^2+^ and Ni^2+^) at 1 mM final concentration. Temperature optima were determined between 20° and 60°C. For kinetic analyses, nucleotide concentrations ranged from 1000 to 0.78 µM.

### Human fibrinogen receptor (GPIIb/IIIa) binding assays

Binding of 5′Nuc/SUMO fusion protein to human GPIIb/IIIa was evaluated by ELISA, largely in the same conditions as described elsewhere [45]. Purified human fibrinogen receptor (AssayPro), was coated at 40 nM in Nunc Maxisorb plates. Binding of 5′Nuc/SUMO (250 – 1.95 nM) onto GPIIb/IIIa was assessed in the presence 0.1% Tween 20, either or not in competition with varying concentrations (20 – 0.05 nM) of human fibrinogen (Sigma), using a polyclonal anti-5′Nuc IgG (1 µg/ml) or an anti-SUMO IgG (1/2000, Lifesensors) followed by detection with a horseradish peroxidase-conjugated anti-rabbit IgG (1/1000, Sigma) and 3,3′,5,5′-tetramethylbenzidine substrate (Sigma). The reaction was stopped by 1∶3 addition of 1 M H_2_SO_4_ and optical densities were measured at 450 nm.

### Human platelet aggregation and disaggregation assays

Venous blood was obtained from healthy volunteers using Monovette coagulation tubes (Sarstedt) resulting in an anticoagulation with 10.6 mM citrate. Platelet-rich plasma (PRP) was obtained as the upper layer after 15 min centrifugation at 150×*g* at 22°C. Platelet-poor plasma (PPP), which served as the 100% transmission baseline, was prepared by pelleting the platelets at 1500×*g* for 15 min. Aggregation of platelets in the presence or absence of total tsetse saliva (0.625–10 µg/ml) and the individual 5′Nuc recombinant protein (10–20 µg/ml) in response to 5 and 10 µM ADP was evaluated at 37°C in a microtiter plate reader (MultiScan Ascent, Thermo). Reduction in optical density (increase in transmission) at 650 nm wavelength was monitored at 15 second intervals, with 60 rpm shaking between each reading, as a measure for platelet aggregation. For disaggregation experiments, human platelets were maximally aggregated with a 10 µM ADP trigger, followed by the addition of saliva (1.25–2.5 µg/ml) or recombinant 5′Nuc/SUMO (10–20 µg/ml) and O.D. measurement at 650 nm.

### *In vivo* RNA interference (RNAi)

For the *in vivo* functional analysis of the 5′nucleotidase, the RNA interference (RNAi) method was applied as described previously [44]. To generate the template for 5′Nuc-specific dsRNA (length: 496 bp) production by *in vitro* transcription (IT), a plasmid containing the full length 5′Nuc coding sequence (pET17b:5′Nuc) was used as PCR template in combination with following primers: 5′Nuc(IT) sense (5′- TAATACGACTCACTATAGGGGCAGACAGCTTGTACGACCA-3′) and 5′Nuc(IT) antisense (5′-TAATACGACTCACTATAGGGTCATGAATTCGATCACGGAA-3′). A control IT template was derived from pBlueScript SK(+) for the production of a control dsRNA as described earlier[44]. Purified IT templates were transcribed using the Megascript RNAi kit (Ambion), following the manufacturer's instructions. DsRNA was further purified as described previously and stored at −20°C until fly injection.

Tsetse flies, 48 h after the last blood meal, were briefly anaesthetised by cold shock and micro-injected intrathoracally with a single dose of 15µg dsRNA. For the evaluation of the transcriptional and translational silencing efficiency, 10 pairs of glands were isolated at eight and twelve days after dsRNA injection and saliva and RNA was purified followed by SDS-PAGE and RT-qPCR. At day 12 after dsRNA injection, feeding efficiencies (blood meal weights and feeding times) of individual flies on F1 (C57Bl/6×Balb/c) mice were monitored within a maximum time of 10 minutes as described previously [43].

## Supporting Information

Table S1 Similarity among selected members of the 5′nucleotidase family. Percentage of sequence identity and similarity (in parenthesis) of the deduced 5′nucleotidase-related protein of the tsetse fly *G. m. morsitans* (AAK63848); Dros5Na and Dros5Nb, putative 5′nucleotidases of *Drosophila melanogaster* (Q9VZ33 and Q9V824); chrysoptin from the deer fly *Chrysops* sp. (Q9U9I6); AgApy/5N, Ag5N and AgApy, different putative apyrase/5′nucleotidase sequences of the mosquito *Anopheles gambiae* (Q8MU75, Q9UB34 and Q9TW03); Ll5N, a putative 5′nucleotidase from the sandfly *Lutzomyia longipalpis* (Q9XZ43); AeApy, *Aedes aegypti* mosquito apyrase (P50635); Bm5N, a 5′nucleotidase from the tick *Boophilus microplus* (P90696); and Hum5N, a 5′nucleotidase from human *Homo sapiens* (P21589).
(0.04 MB DOC)

Table S2 5′Nuc inhibitor profile. The ATP(D)ase activity of recombinant 5′Nuc was assessed in the presence of a panel of ATPase inhibitors. Indicated are the used inhibitor concentrations and the percentual activity with standard errors as compared to the control setting.
(0.03 MB DOC)

Figure S1 5′Nuc specific silencing by RNA interference. Relative normalized *5′nuc* and *sgp3* expression levels at days 8 and 12 in control RNAi and 5′Nuc RNAi treated flies as determined by RT-qPCR. Percentages 5′Nuc silencing are indicated above the respective bars.
(1.25 MB TIF)
