# Supplementary material for: Identification of a Tsetse Fly Salivary Protein with Dual Inhibitory Action on Human Platelet Aggregation
Source: PLoS One. 2010 Mar 23;5(3):e9671. doi: 10.1371/journal.pone.0009671 (PMC2843633; doi:10.1371/journal.pone.0009671)
Supplement: Table S1 — Similarity among selected members of the 5′nucleotidase family. Percentage of sequence identity and similarity (in parenthesis) of the deduced 5′nucleotidase-related protein of the tsetse fly G. m. morsitans (AAK63848); Dros5Na and Dros5Nb, putative 5′nucleotidases of Drosophila melanogaster (Q9VZ33 and Q9V824); chrysoptin from the deer fly Chrysops sp. (Q9U9I6); AgApy/5N, Ag5N and AgApy, different putative apyrase/5′nucleotidase sequences of the mosquito Anopheles gambiae (Q8MU75, Q9UB34 and Q9TW03); Ll5N, a putative 5′nucleotidase from the sandfly Lutzomyia longipalpis (Q9XZ43); AeApy, Aedes aegypti mosquito apyrase (P50635); Bm5N, a 5′nucleotidase from the tick Boophilus microplus (P90696); and Hum5N, a 5′nucleotidase from human Homo sapiens (P21589). (0.04 MB DOC) [file pone.0009671.s001.doc]

|  | Dros  5N_a_ | Chrysoptin | Ag  Apy/5N | Ag  5N | Ag  Apy | Ll  5N | Ae  Apy | Dros  5N_b_ | Bm  5N | Hum  5N |
| --- | --- | --- | --- | --- | --- | --- | --- | --- | --- | --- |
| Gmm5N (555aa) | 43 (63) | 38 (62) | 36 (56) | 34 (52) | 33 (52) | 34 (51) | 33 (52) | 31 (49) | 28 (48) | 30 (49) |
| Dros5N_a_ (557aa) |  | 44 (67) | 41 (60) | 35 (53) | 33 (50) | 34 (52) | 31 (53) | 31 (49) | 30 (50) | 29 (49) |
| Chrysoptin (554aa) |  |  | 41 (62) | 32 (54) | 33 (54) | 34 (54) | 34 (54) | 29 (49) | 32 (52) | 33 (52) |
| AgApy/5N (568aa) |  |  |  | 35 (51) | 35 (52) | 35 (50) | 31 (48) | 31 (48) | 31 (47) | 30 (47) |
| Ag5N (570aa) |  |  |  |  | 45 (64) | 29 (48) | 56 (70) | 26 (48) | 26 (46) | 29 (49) |
| AgApy (557aa) |  |  |  |  |  | 47 (64) | 28 (46) | 27 (45) | 28 (44) | 31 (48) |
| Ll5N (572 aa) |  |  |  |  |  |  | 28 (47) | 43 (59) | 35 (52) | 40 (57) |
| AeApy (562aa) |  |  |  |  |  |  |  | 27 (47) | 27 (45) | 28 (47) |
| Dros5N_b_ (599aa) |  |  |  |  |  |  |  |  | 30 (46) | 35 (52) |
| Bm5N (580aa) |  |  |  |  |  |  |  |  |  | 36 (54) |
| Hum5N (574aa) |  |  |  |  |  |  |  |  |  |  |
