# Supplementary material for: Identification of a Tsetse Fly Salivary Protein with Dual Inhibitory Action on Human Platelet Aggregation
Source: PLoS One. 2010 Mar 23;5(3):e9671. doi: 10.1371/journal.pone.0009671 (PMC2843633; doi:10.1371/journal.pone.0009671)
Supplement: Table S2 — 5′Nuc inhibitor profile. The ATP(D)ase activity of recombinant 5′Nuc was assessed in the presence of a panel of ATPase inhibitors. Indicated are the used inhibitor concentrations and the percentual activity with standard errors as compared to the control setting. (0.03 MB DOC) [file pone.0009671.s002.doc]

| **Inhibitors** | **Concentration** | **ATPase (% activity ± SE)** | **ADPase (% activity ± SE)** |
| --- | --- | --- | --- |
| Ouabain | 1 mM | 102 ± 10 | 85 ± 8 |
| Levamisol | 1 mM | 113 ± 12 | 110 ± 13 |
| AP5A | 1 mM | 83 ± 12 | 72 ± 10 |
| Adenosine | 1 mM | 63 ± 7 | 52 ± 7 |
| AMP | 1 mM | 65 ± 4 | 66 ± 3 |
| Concanavalin A | 25 µg/ml | 191 ± 14 | 129 ± 13 |
| DIDS | 100 µM | 34 ± 7 | 58 ± 12 |
| Sodium vanadate | 1 mM | 77 ± 9 | 66 ± 8 |
| Sodium azide | 10 mM | 83 ± 7 | 83 ± 9 |
| Sodium fluoride | 10 mM | 8 ± 1 | 3 ± 2 |
| DEPC | 2 mM | 22 ± 1 | 3 ± 2 |
