# Supplementary figures and images for: Identification of a Tsetse Fly Salivary Protein with Dual Inhibitory Action on Human Platelet Aggregation
Source: PLoS One. 2010 Mar 23;5(3):e9671. doi: 10.1371/journal.pone.0009671 (PMC2843633; doi:10.1371/journal.pone.0009671)

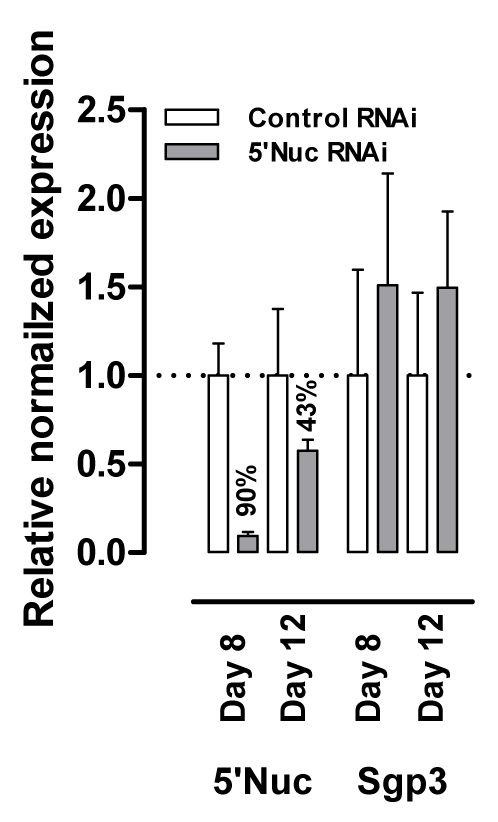

Supplement: Figure S1 — 5′Nuc specific silencing by RNA interference. Relative normalized 5′nuc and sgp3 expression levels at days 8 and 12 in control RNAi and 5′Nuc RNAi treated flies as determined by RT-qPCR. Percentages 5′Nuc silencing are indicated above the respective bars. (1.25 MB TIF) [file pone.0009671.s003.tif]
